# Supplementary material for: Rapid synthesis of micron-thick flexible graphite films via non-equilibrium carbon flux engineering
Source: Nat Commun. 2026 Feb 28;17:3280. doi: 10.1038/s41467-026-70028-8 (PMC13066362; doi:10.1038/s41467-026-70028-8)
Supplement: Supplementary file 1 — Supplementary Information [file 41467_2026_70028_MOESM1_ESM.pdf]

Supplementary Information for

Rapid synthesis of micron-thick flexible graphite films via non-equilibrium carbon flux engineering

Haiyang Liu[1,2,†], Zhen Wang[2,†], Xu Wang[1,2,†], Jiayu Chang[2], Xinfu Hou[3], Linxuan Li[4], Mengyuan Liu[5], Xiongzhi Zeng[6], Qi Cai[1,2], Qingyu Zhou[2], Junwei Deng[2,7], Chengjin Wu[2], Sicong Zheng[2,7], Zhenyu Li[6], Mengxi Liu[5], Wu Zhou[4], Bo Sun[3,*], Luzhao Sun[1,2,*], Zhongfan Liu[1,2,*]

[1]Center for Nanochemistry, Beijing Science and Engineering Center for Nanocarbons, Beijing National Laboratory for Molecular Sciences, College of Chemistry and Molecular Engineering, Peking University, Beijing 100871, China

[2]Technology Innovation Center of Graphene Metrology and Standardization for State Market Regulation, Beijing Graphene Institute, Beijing, 100095, China

[3]Tsinghua SIGS, Tsinghua University, Shenzhen 518055, China

[4]School of Physical Sciences, University of Chinese Academy of Sciences, Beijing, 100049, China

[5]CAS Key Laboratory of Standardization and Measurement for Nanotechnology, National Center for Nanoscience and Technology, Beijing 100190, China

[6]Key Laboratory of Precision and Intelligent Chemistry, University of Science and Technology of China, Hefei, 230026, China

[†]These authors contributed equally to this work.
*Corresponding author. Email: sun.bo@sz.tsinghua.edu.cn (B.S.); sunlz-cnc@pku.edu.cn (L.S.); zfliu@pku.edu.cn (Z.L.)

Content

Supplementary Note 1 to Note 4

Supplementary Table 1 and Table 2

Supplementary Fig. 1 to Fig. 16

Supplementary Reference 1-7

Supplementary Notes

Supplementary Note 1. Theoretical derivation of the diffusion mechanism of carbon within the Ni bulk phase

To quantitatively evaluate the transport rate of carbon within the Ni bulk phase, a theoretical model was established based on Fick's laws of diffusion. Considering that carbon diffusion in nickel predominantly followed interstitial and grain boundary diffusion mechanisms, the Ni substrate was simplified as an ideal face-centered cubic (FCC) lattice, and only the interstitial diffusion pathway in a single crystal was considered for the calculation. According to Fick's First Law, the steady-state diffusion flux J ($kg \cdot m^{-2} \cdot s^{-1}$) was related to the concentration gradient：

$$J = -D\frac{\partial C}{\partial x} \quad (1)$$

The parameter D ($m^2 \cdot s^{-1}$) was defined as the diffusion coefficient, and C ($kg \cdot m^{-3}$) represented the mass concentration of carbon. In practice, the non-steady-state diffusion process was required to be described by Fick's second law：

$$\frac{\partial C}{\partial t} = D\left(\frac{\partial^2 C}{\partial x^2} + \frac{\partial^2 C}{\partial y^2} + \frac{\partial^2 C}{\partial z^2}\right) = D\,\nabla^2 C \quad (2)$$

Its error function solution was expressed as follows:

$$\frac{C_s - C}{C_s - C_0} = erf\left(\frac{x}{2\sqrt{Dt}}\right) \quad (3)$$

Here, C_S represented the surface concentration, while C_0 denoted the initial bulk concentration. For a nickel foil with a thickness of 50 μm (*i.e.,* diffusion distance x = 50 μm), assuming $C_0 = 0$ and the concentration at the opposite side reached $0.5C_S$ after time t, the following relation was obtained by substituting into the error function solution:

$$erf\left(\frac{x}{2\sqrt{Dt}}\right) = 0.5 \quad (4) \Rightarrow \frac{x}{2\sqrt{Dt}} = 0.521 \quad (5)$$

The diffusion coefficient equation for carbon in nickel proposed by Lander et al. was adopted[1].

$$lnD = 0.909 - \frac{20200}{T} \quad (6)$$

At the growth temperature $T = 1573$ K, the diffusion coefficient D was calculated to be 6.571×10^{-6} $cm^2 \cdot s^{-1}$. By solving the coupled equations, the diffusion time t was determined to be approximately 3.8 s. This result indicated that, at 1300 °C, carbon atoms could penetrate a 50 μm-thick nickel foil within only a few seconds. Although the actual growth rate was also constrained by nucleation, layer formation, and stacking kinetics, such efficient bulk diffusion confirmed that

high carbon-solubility metals particularly polycrystalline nickel, where grain boundaries could further accelerate transport offered significant advantages for the rapid synthesis of graphite films. Within the temperature range of 700–1300 °C, the solubility of carbon in nickel can be described by the following equations:

$$ln\, S = 2.48 - \frac{4880}{T} \quad (7)$$

$$S_P = S_{P0} \exp\left(\frac{H_P}{kT}\right) \quad (8)$$

where S denotes the solubility. In Equation (7), S is expressed as the mass of carbon per 100 g of nickel (g/100 g), whereas in Equation (8), S^P is expressed as the number of carbon atoms per cubic centimeter (atoms/cm^3).

In the present experiment, at a set temperature of T = 1573 K, the measured solubilities were: S = 0.5368 g/100 g, $S^P = 2.405 \times 10^{21}$ atoms/cm^3. The volume of the nickel foil used in the experiment was 2×10^{-2} cm^3 (dimensions: 2 cm × 2 cm × 50 μm), with an average mass of 0.1821 g. Based on Equation (7), the theoretical carbon solubility limit was calculated to be 0.000977 g, whereas the value calculated from Equation (8) was 0.000958 g, giving a relative error of approximately 2%. Assuming complete carbon precipitation and subsequent graphite formation, the theoretical thickness of the graphite layer was estimated to be H = 1.17 μm.

Supplementary Note 2. Calculation of the growth rate of graphite films

The graphite films were successfully synthesized on nickel foil substrates via the Joule heating-induced carburization (PJHIC) method. To accurately characterize the film thickness, White Light Interferometry (WLI) was employed to measure samples obtained at various growth durations t (in minutes). To ensure statistical reliability and to assess surface uniformity, no fewer than four representative surface regions were selected for each time point. The reported graphite thickness, denoted as δ (in nanometers), was calculated as the arithmetic mean of all individual measurements, and its variability was represented by the standard deviation (SD), expressed as δ ± SD nm. This SD value was also used to evaluate both the measurement reproducibility and the uniformity of the film surface. Given that the core kinetics of the PJIC process involve Joule heating–driven solid-state carbon diffusion and graphitization, the growth rate v (in nm·min^{-1}) was defined as a key efficiency metric. v was calculated as the ratio of the accumulated film thickness δ (obtained from WLI measurements, averaged over $n \geq 4$ regions, in nm) to the cumulative effective PJIC duration t (in min). Here, the effective treatment time t referred specifically to the total duration during which the temperature exceeded 1000 °C.

$$v = \frac{\delta}{t} \quad (9)$$

Supplementary Note 3. two-stage model for non-equilibrium segregation process

A two-stage model that quantitatively describes the non-equilibrium segregation process, emphasizing the roles of interfacial carbon supersaturation (ΔC) and phase transformation driving force ($\Delta\mu$).

Stage I: Solute depletion-driven carbon segregation to the interface

The PJHIC process initiates with rapid cooling from a starting high temperature (T_0, 1300°C) to a lower segregation temperature (1000°C < T < 1300°C). This creates a massive transient supersaturation at the metal-graphite interface. According to the Ni–C phase diagram and the empirical relationship (Equation 7) proposed by Lander & Marshall[1], The rapid cooling time within 4.0~4.7 seconds contributes great interfacial supersaturation $\Delta C = C_0(1300°C) - C_1(1000°C)$ = 0.277 wt.%. Note that, for small values of S, the dimensionless mass fraction C can be directly calculated by $C = \frac{S}{100+S} \approx \frac{S}{100} = \frac{1}{100}\exp\left(2.480 - \frac{4880}{T}\right)$.

The ΔC drives a chemical potential difference $\Delta\mu$. The molar Gibbs free energy (μ) is expressed as:

$$\mu(C,T) = \mu^0(T) + k_B T \ln(C) \quad (10)$$

where $\mu^0(T)$ is the standard (reference-state) chemical potential of carbon in Ni at temperature T, k_B is the Boltzmann constant. We assume that the carbon has been solved in Ni bulk at T_0 = 1300 °C with an equilibrium carbon solubility $C_0(1300°C)$ = 0.536 wt.%. When the temperature drops, the **excess chemical potential** that drives segregation to the surface at temperature T is:

$$\Delta\mu = \mu(C_0, T) - \mu(C(T), T) = k_B T \ln\frac{C_0}{C(T)} \quad (11)$$

For T_1 = 1000°C = 1273 K, $\Delta\mu \approx 7.73 \times 10^3\, J/mol\ (\approx 0.080\, eV/mol)$. The significantly high ΔC and $\Delta\mu$ promote the subsequent phase transformation from carbon atoms to graphite layers.

Stage II: Supersaturation-driven graphite layer formation

The supersaturated carbon at the interface must overcome the nucleation barrier to form graphite layers. In classical nucleation theory, graphene nucleation on metal surfaces is an activated process in which a stable nucleus forms once it surpasses a critical size, balancing the energetic competition between creating a new interface (surface energy cost) and forming a new phase (volumetric energy gain from supersaturation). A new phase (graphene nucleus) forms via

discrete nucleation events rather than continuous transformation. Nuclei are assumed circular (2D disc-shaped) and energetically isotropic. Interface properties (line energy λ) and driving force ($\Delta\mu$) are considered constant and uniform during nucleation. Nucleation is thermodynamically driven and kinetically activated, involving the random fluctuation-driven formation of critical-sized clusters[2,3].

The Gibbs free energy for forming a circular graphene nucleus of radius *r* is:

$$\Delta G(r) = 2\pi r\lambda - \pi r^2 \rho_g \Delta\mu \qquad (12)$$

Where $\lambda \approx 1\ \mathrm{eV/nm}$ is the graphene-Ni line energy[4], $\rho_g = 3.82 \times 10^{19}\ atom\ m^{-2}$ is the areal atomic density, and $\Delta\mu$ is the areal thermodynamic driving force. The critical nucleation radius r^* and barriers ΔG^* follow from $\partial\Delta G/\partial r = 0$:

$$r^* = \frac{\lambda}{\rho_g \Delta\mu},\ \ \Delta G^* = \frac{\pi\lambda^2}{\rho_g \Delta\mu} \qquad (13)$$

We can find that the r^* and ΔG^* both are inversely proportional to $\Delta\mu$. The high $\Delta\mu$ in our PJHIC process significantly lowers ΔG*, facilitating high nucleation density and rapid layer formation (Fig. 1g,h). For nucleation of subsequent layers (2nd layer, 3rd layer,…) beneath the first layer, the same theoretical framework applies, though with a generally lower interfacial line energy λ_2 due to spatial confinement and weaker coupling to metal substrate. Graphene thickening proceeds once the interfacial carbon activity sufficiently reduces ΔG^* small enough.

Comparison between PJHIC and conventional isothermal CVD

In conventional isothermal CVD at 1000 °C, supersaturation is limited and originates not from bulk solubility changes, but from the surface decomposition of hydrocarbon precursors (e.g., methane, acetylene). These precursors adsorb and dissociate to form active carbon species, which then migrate to create localized supersaturation. Given this fundamental difference in mechanism, ***carbon atomic flux (F)*** provides a more direct and quantitative parameter for comparing the mass transport behaviors of CVD and PJHIC processes.

For the PJHIC process, the effective growth duration is within 4s ~ 4.7s, and the vertical growth rate is calculated as 11,162.8 nm min^{-1} (see Response to Comment 4). The carbon flux is calculated as:

$$F_{PJHIC} = 2.1 \times 10^{22}\ atoms/(m^2 \cdot s) \qquad (14)$$

The local concentration of active carbon species in CVD has not been experimentally quantified. So we estimated the effective flux (for lattice incorporation) using data from two

benchmark CVD studies: I) the current growth record-holder[5], II) a high-speed process on CuNi alloy[6].

$$F_{\mathrm{I}} = 3.06 \times 10^{20} atoms/(m^2 \cdot s) \quad (15)$$

$$F_{\mathrm{II}} = 1.27 \times 10^{17} atoms/(m^2 \cdot s) \quad (16)$$

The calculation results clearly demonstrate that the carbon atomic flux of the PJHIC process (2.11×10^{22} atoms m^{-2} s^{-1}) exceeds that of conventional isothermal CVD processes (3.06×10^{20} atoms $m^{-2}s^{-1}$) by at least two orders of magnitude, highlighting the exceptional mass transport efficiency and high segregation driving force enabled by the non-equilibrium forced segregation mechanism.

Supplementary Note 4. The single segregation limit of graphite films

By systematically increasing the PMMA coating volume (0.2-0.8 mL) and extending the annealing time to 5 minutes, a maximum experimental thickness of 1.067 ± 0.032 μm was achieved at a coating volume of 0.6 mL. The relationship between film thickness and PMMA dosage exhibited a non-monotonic trend, first increasing and then decreasing. This behavior was attributed to excessive PMMA decomposition, which generated gaseous byproducts (CH_4, C_2H_4, CO, etc.), resulting in substantial carbon loss and thus limiting further thickness growth. Details regarding carbon loss of PMMA please see in Supplementary Table 1.

Supplementary Table

Supplementary Table 1 | Mass change under different PMMA coating amounts.

Number	m_{Ni}/g	V_{PMMA}/mL	$m_{PMMA/Ni}$/g	Δm_{PMMA} (g)	$m_{Gr/Ni}$/g	Δm_{Gr}/g	The carbon loss rate/%	The average/ %	The error/ %
1	0.1839	0.2000	0.1941	0.0102	0.1852	0.0013	12.75%		
2	0.1801	0.4000	0.1994	0.0193	0.1822	0.0021	10.88%		
3	0.1855	0.6000	0.2177	0.0322	0.1874	0.0019	5.90%	7.42%	3.73%
4	0.1836	0.8000	0.2232	0.0396	0.1849	0.0013	3.28%		
5	0.1778	1.0000	0.2289	0.0511	0.1800	0.0022	4.31%		

Supplementary Table 2 | The parameters used in the thermal diffusion model[7] to extract the thermal conductivity.

Parameter	Value
Volumetric heat capacity of Al (C_{Al})	2.42 $Jcm^{-3}K^{-1}$
Thermal conductivity of Al (Λ_{Al})	170 $Wm^{-1}K^{-1}$
Thickness of Al (h_{Al})	109 nm
Volumetric heat capacity of Graphite ($C_{Graphite}$)	1.58 $Jcm^{-3}K^{-1}$
$1/e^2$ radius of the laser (w_0)	12 μm / 3 μm

Supplementary Figures

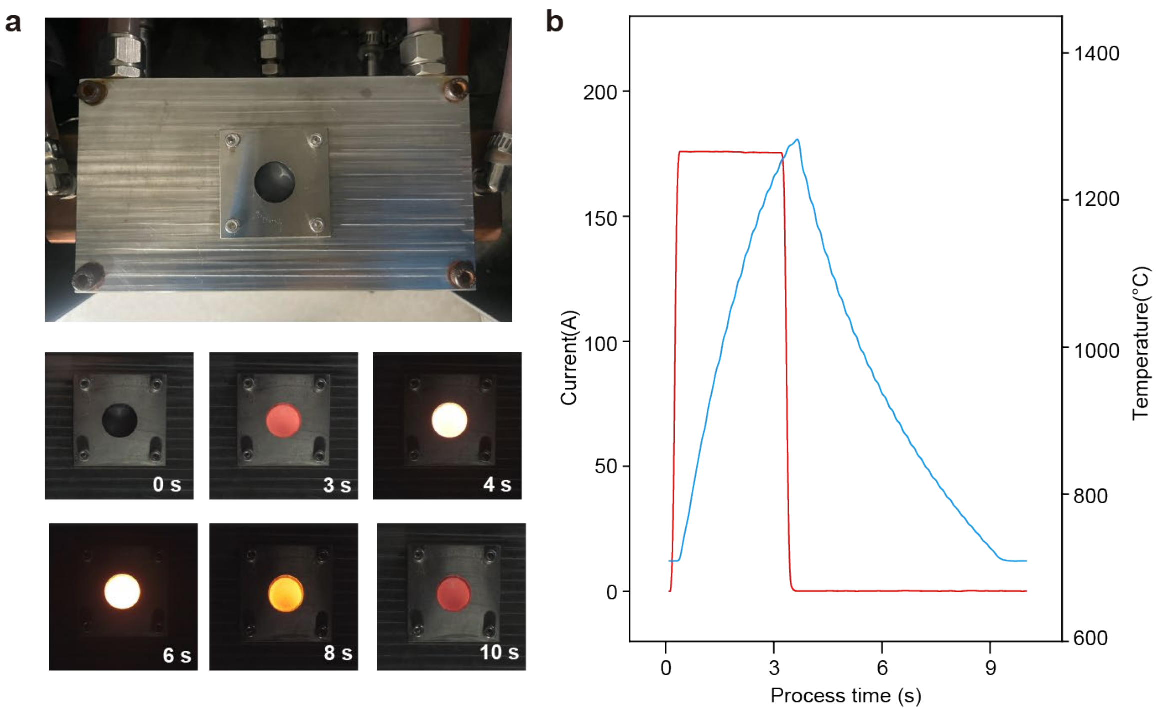

Supplementary Fig. 1| Pulsed Joule heating equipment. (a) Photographs at different heating durations: 0 s, 3 s, 4 s, 6 s, 8 s, and 10 s. (b) Typical heating profiles of the PJHIC process.

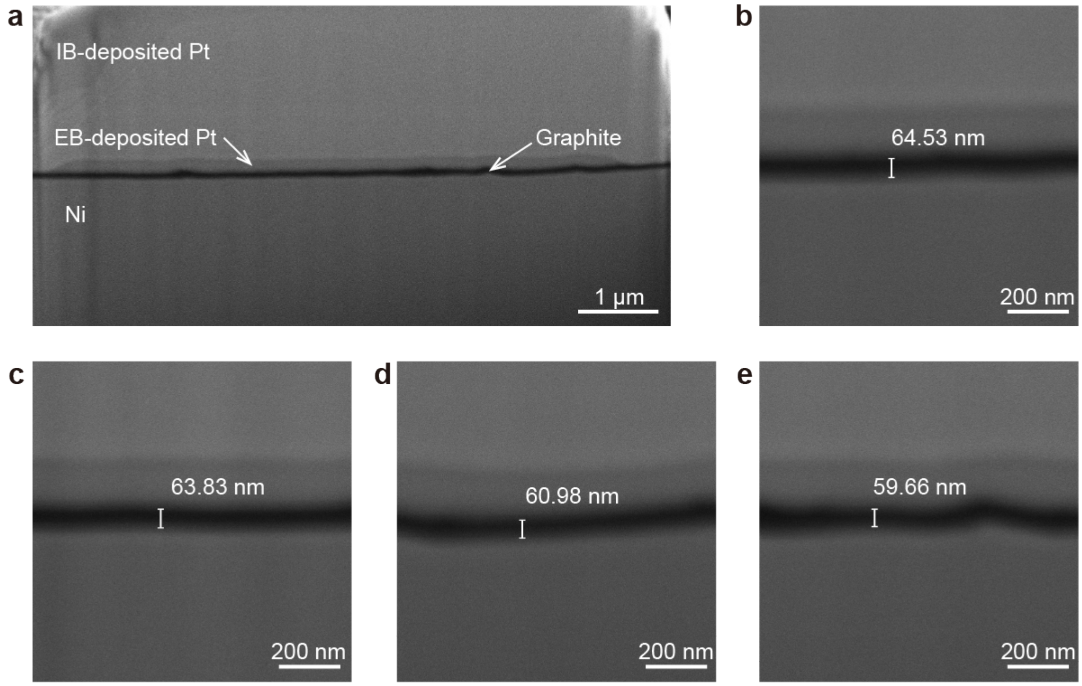

Supplementary Fig. 2 | Thickness characterization of as-obtained graphite with growth time of 11 s via cross-sectional SEM. (a) SEM image of the FIB-prepared cross-section specimen. (b-e) SEM images showing graphite thickness measurements at four different positions.

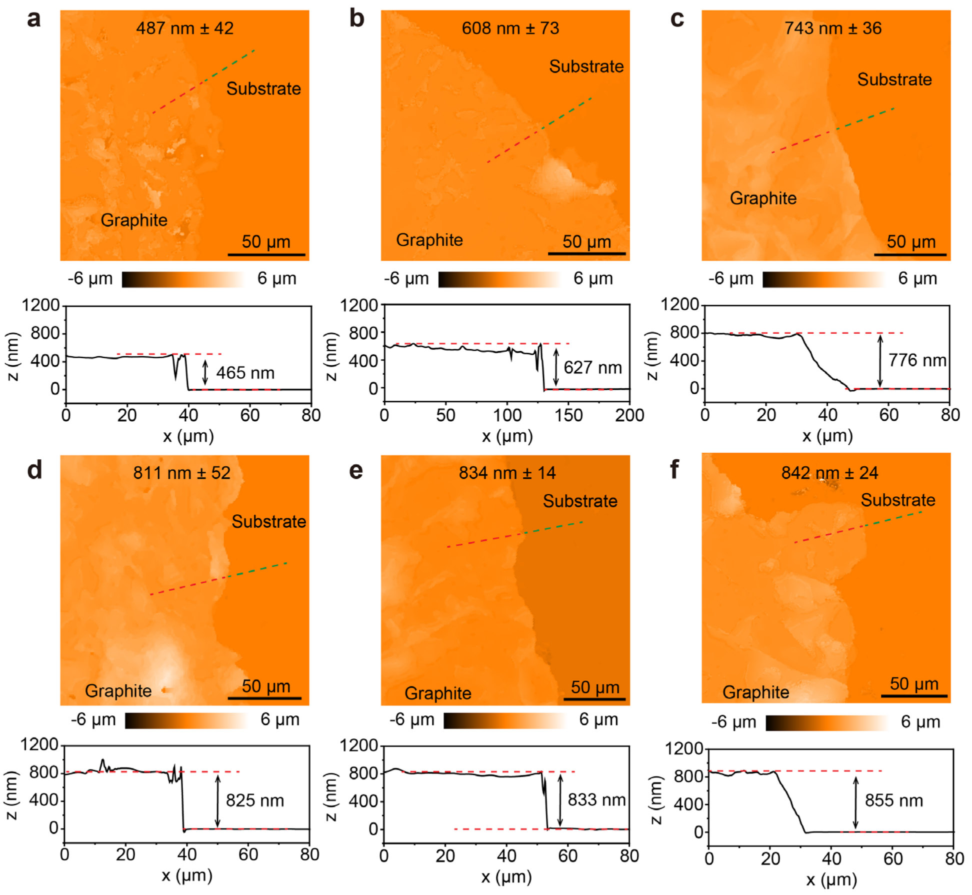

Supplementary Fig. 3 | Thickness characterization of graphite edges. (a-f) WLI scanning topography images and their corresponding cross-sectional height curves of graphite film edges with growth durations of 42 s (a), 51 s (b), 61 s (c), 72 s (d), 102 s (e), and 132 s (f). The error bars represent the standard deviation of repeated experiments.

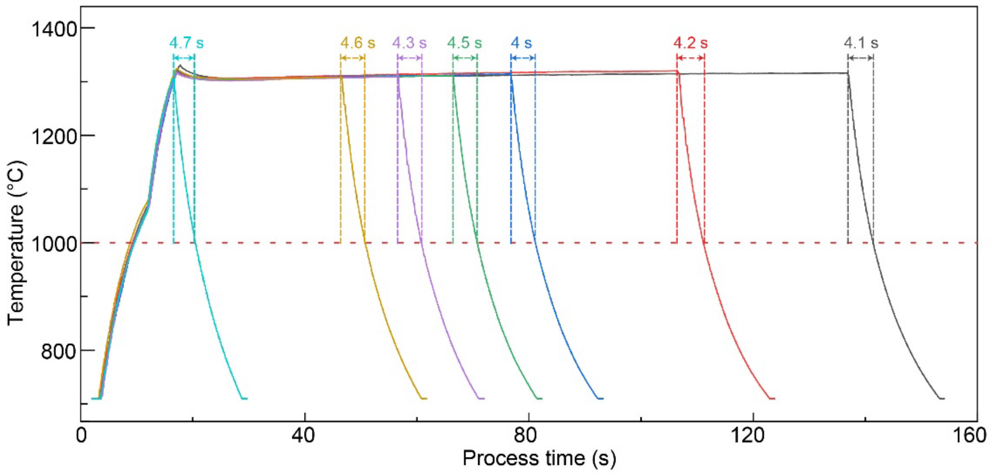

Supplementary Fig. 4 | Analysis of cooling duration and corresponding instantaneous segregation rates. The specific cooling time windows (from 1300 °C to 1000 °C) associated with the temperature profiles in Fig. 1f are delineated. the instantaneous segregation rates are calculated to be: v_1 = 792 nm/min, v_2 = 6,354 nm/min, v_3 = 8,484 nm/min, v_4 = 9,906 nm/min, v_5 = 12,168 nm/min, v_6 = 11,916 nm/min, and v_7 = 12,324 nm/min.

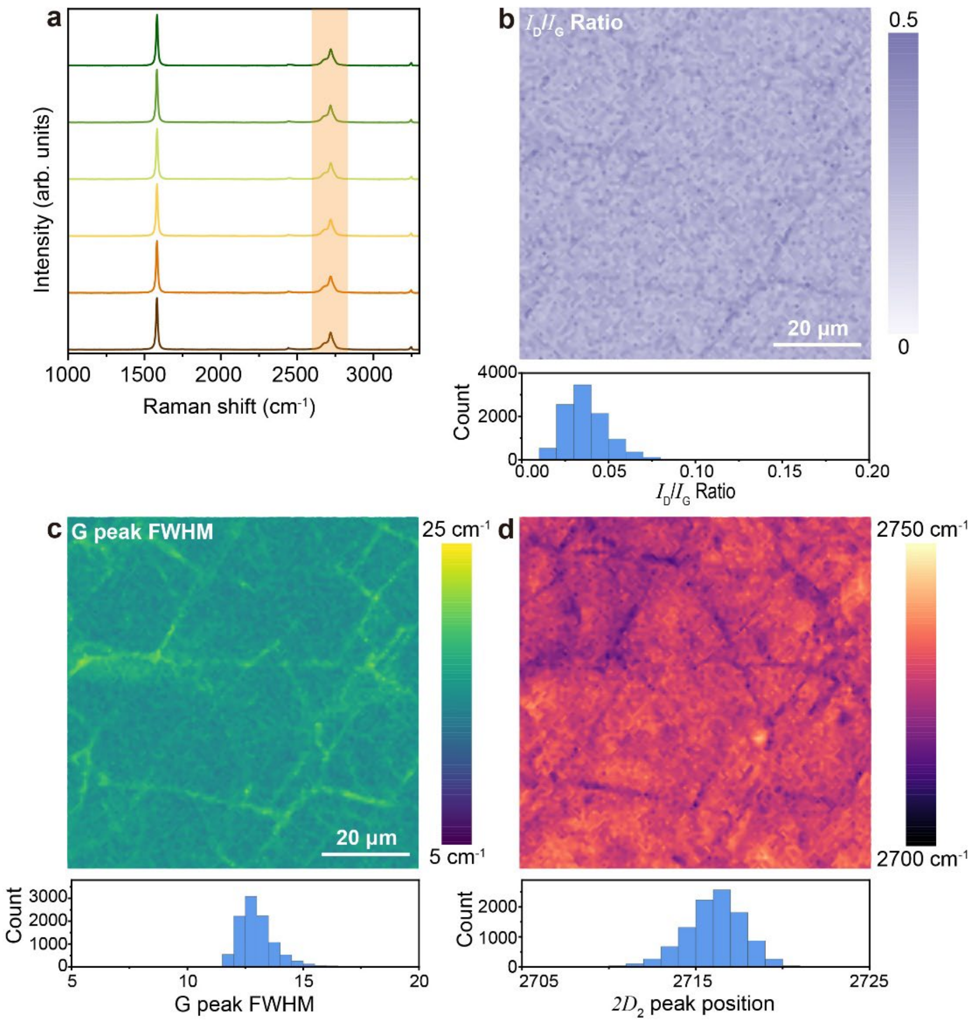

Supplementary Fig. 5 | Raman characterization of graphite films. (a) Typical Raman spectra of the graphite film at 6 random positions. (b-d) Raman mapping of the graphite film showing I_D/I_G (b), FWHM of the G band (c), and peak position of $2D_2$ (d).

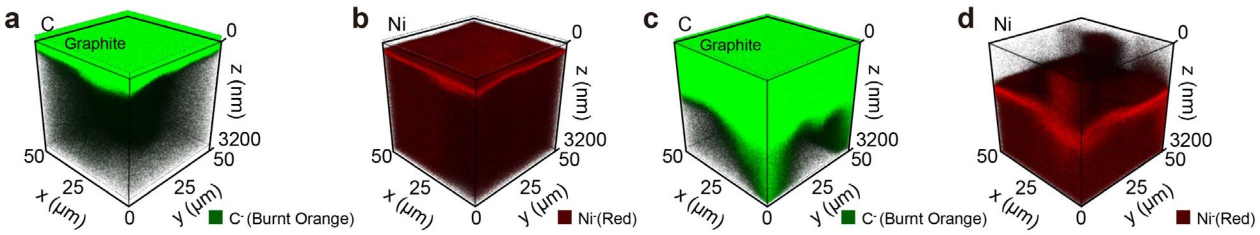

Supplementary Fig. 6 | TOF-SIMS characterization of graphite samples with different growth durations. (a-b) 3D mapping of C element signal intensity (a) and Ni element signal intensity (b) with a growth duration of 12 s. (c-d) 3D mapping of C element signal intensity (c) and Ni element signal intensity (d) with a growth duration of 72 s.

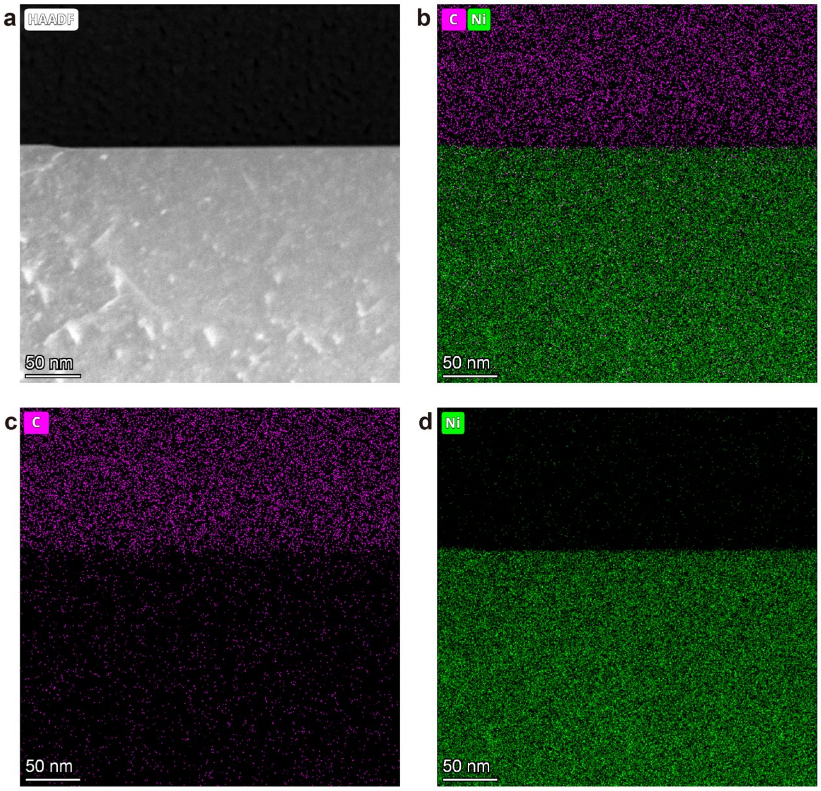

Supplementary Fig. 7 | Element distribution in the cross-section of the sample grown for 72 seconds, characterized by EDS. (a) HAADF image. (b) Element distribution mapping of C and Ni. (c) Element distribution mapping of C. (d) Element distribution mapping of Ni.

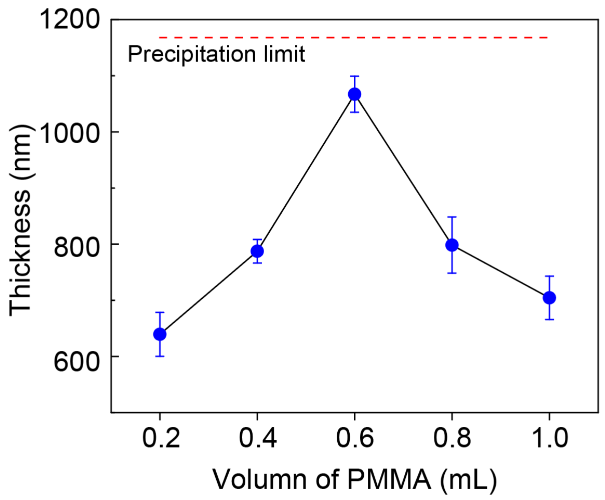

Supplementary Fig. 8 | Variation in the maximum precipitation thickness of graphite films with different PMMA coating amounts. The error bars represent the standard deviation of repeated experiments.

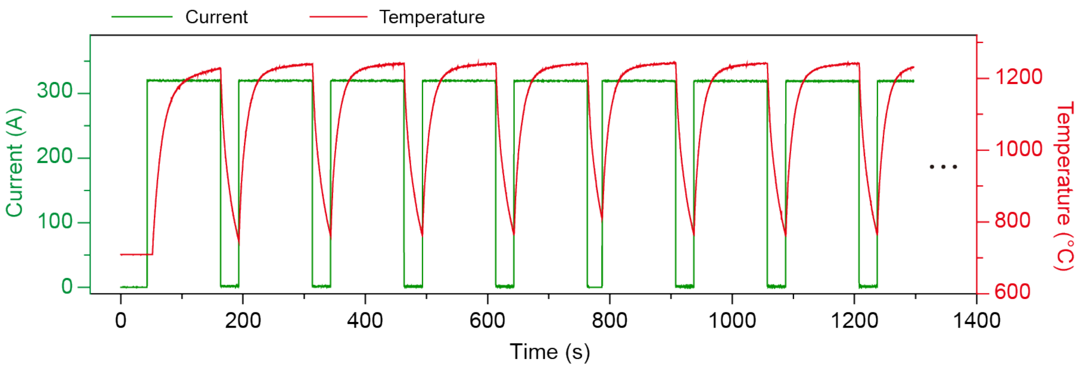

Supplementary Fig. 9 | The curve of current and temperature varying with time during the cyclic heating and cooling process.

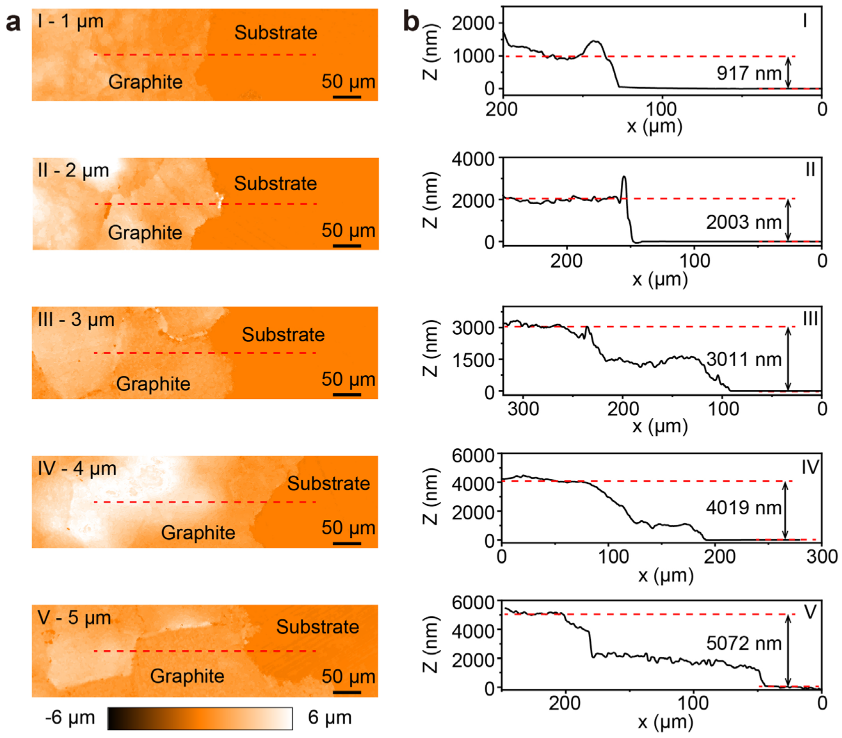

Supplementary Fig. 10 | Graphite films of varying thicknesses segregated on Co foils. (a) White light interferometry surface profiles at the film edges for graphite layers with thicknesses ranging from 1 to 5 μm. (b) Cross-sectional height profiles extracted from white light interferometry scans of graphite film edges with different thicknesses.

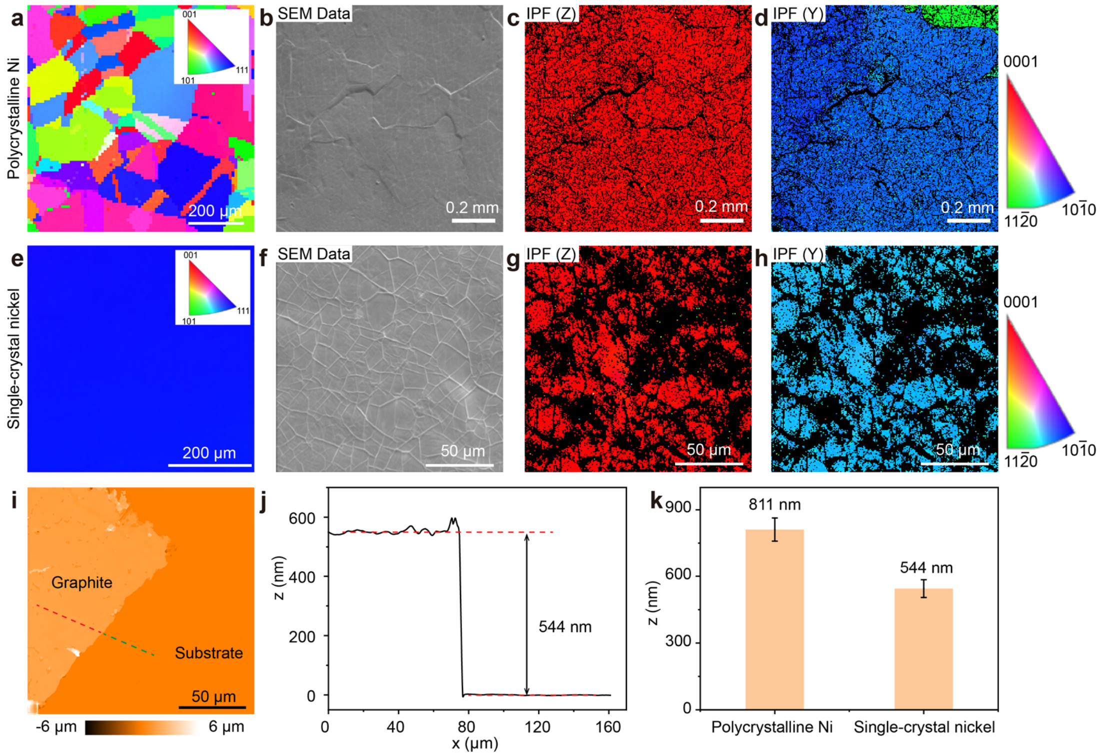

Supplementary Fig. 11 | Grain size of graphite films. (a) EBSD inverse pole figure mapping along the Z-direction of annealed ordinary commercial nickel foils. (b) SEM of ordinary commercial nickel foils. (c, d) EBSD inverse pole figure mappings along the Z-direction and Y-direction of graphite films grown by segregation on the surface of ordinary commercial nickel foils. (e) EBSD inverse pole figure mapping along the Z-direction of single-crystal Ni(111) foils. (f) SEM of ingle-crystal Ni(111) foils. (g h) EBSD inverse pole figure mappings along the Z-direction and Y-direction of graphite films grown by segregation on the surface of single-crystal Ni(111) foils. (i) White light interferometry surface profiles of the film edges of graphite layers on single-crystal nickel foils. (j) Cross-sectional height profiles extracted from white light interferometry scans of the edges of graphite films on single-crystal nickel foils. (k) Comparison of graphite film thicknesses on polycrystalline nickel foils vs. single-crystal nickel foils. The error bars represent the standard deviation of repeated experiments.

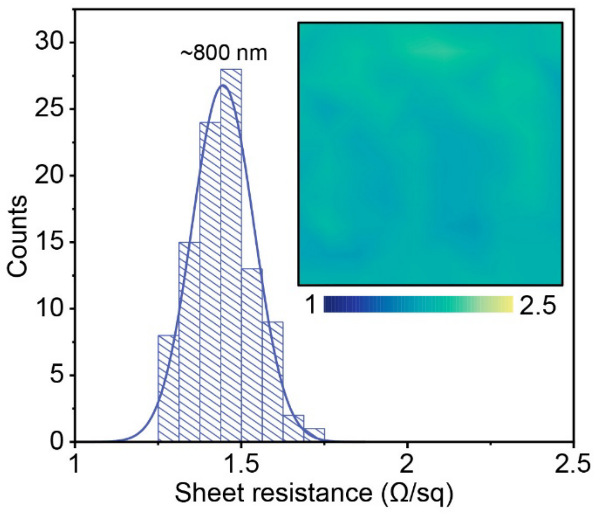

Supplementary Fig. 12 | Statistical analysis of the sheet resistance. Inset: Sheet resistance mapping of graphite films. The electrical conductivity (σ) of the material is related to its sheet resistance (R_s) and thickness (*t/nm*) by the following relation: $\sigma = \frac{1}{R_S\, t}$.

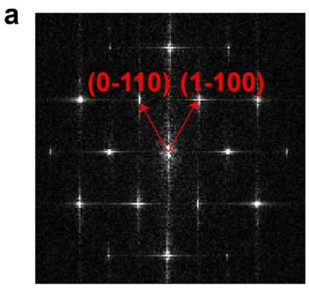

b

	Experimental Value	Theoretical value
0-110	4.65 nm^{-1}	4.70 nm^{-1}
1-100	4.72 nm^{-1}	4.70 nm^{-1}
Included angle	60.1°	60.0°

Supplementary Fig. 13 | FFT analysis of ADF-STEM image. (a) FFT results corresponding to Fig.4f. (b) Comparison of experimentally measured and theoretically simulated lattice constants. The simulation parameters were as follows: sample thickness of 20 nm, electron beam accelerating voltage of 200 kV, convergence semi-angle of 32 mrad, and collection angle range of 36–147 mrad.

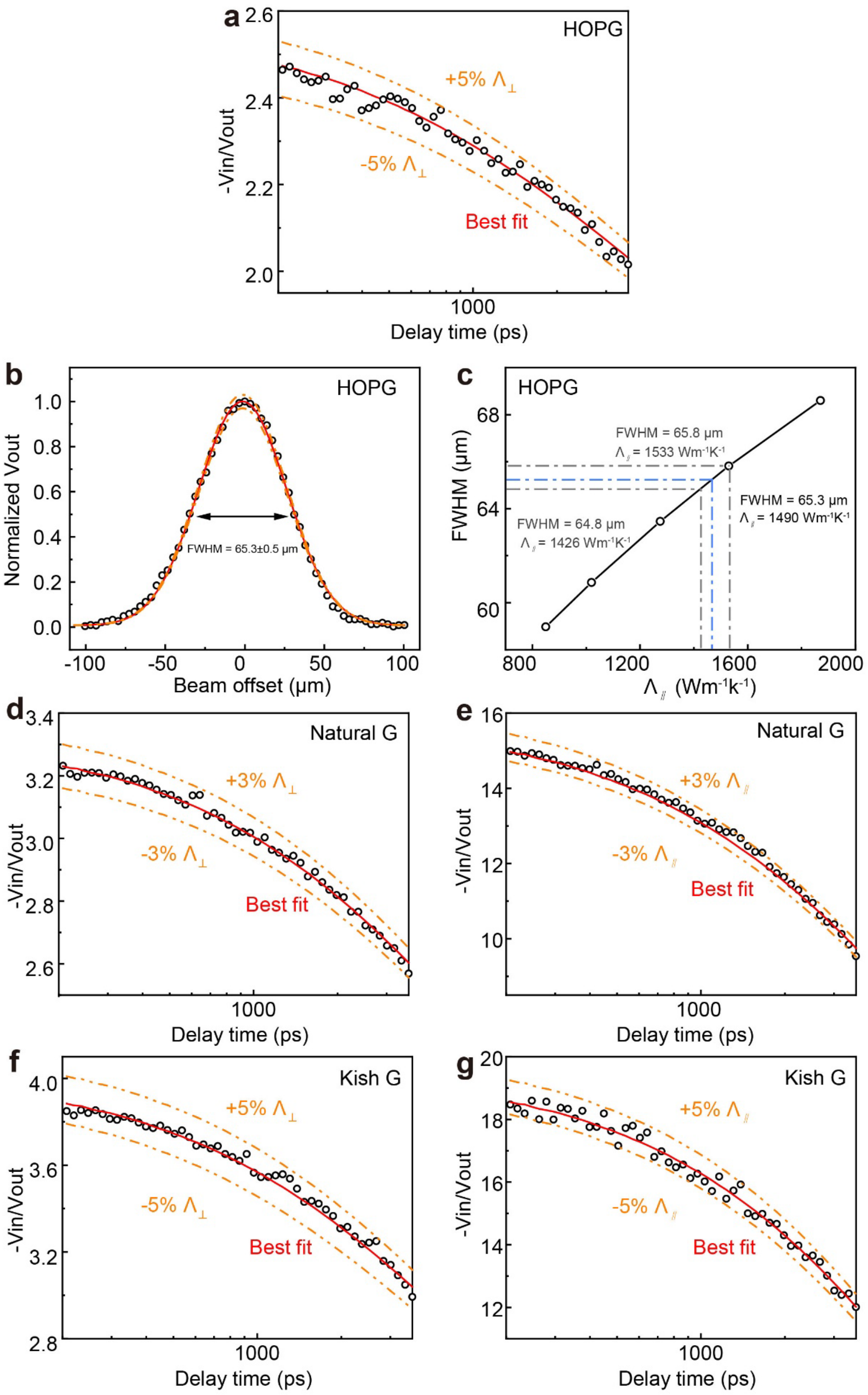
a
HOPG
+5% Λ⊥
-5% Λ⊥
Best fit
-Vin/Vout
Delay time (ps)
b
HOPG
Normalized Vout
FWHM = 65.3±0.5 μm
Beam offset (μm)
c
HOPG
FWHM = 65.8 μm
Λ// = 1533 Wm-1K-1
FWHM = 65.3 μm
Λ// = 1490 Wm-1K-1
FWHM = 64.8 μm
Λ// = 1426 Wm-1K-1
FWHM (μm)
Λ// (Wm-1k-1)
d
Natural G
+3% Λ⊥
-3% Λ⊥
Best fit
e
Natural G
+3% Λ//
-3% Λ//
Best fit
f
Kish G
+5% Λ⊥
-5% Λ⊥
Best fit
g
Kish G
+5% Λ//
-5% Λ//
Best fit

Supplementary Fig. 14 | TDTR data and fitting analysis of HOPG, natural graphite and Kish graphite. (a)Measurement of the cross-plane thermal conductivity of HOPG. (b-c) Beam-offset TDTR measurement of the in-plane thermal conductivity of HOPG. Measurement of the cross-plane (d) and in-plane thermal (e) conductivity of Natural Graphite. Measurement of the cross-plane (f) and in-plane thermal (g) conductivity of Kish graphite.

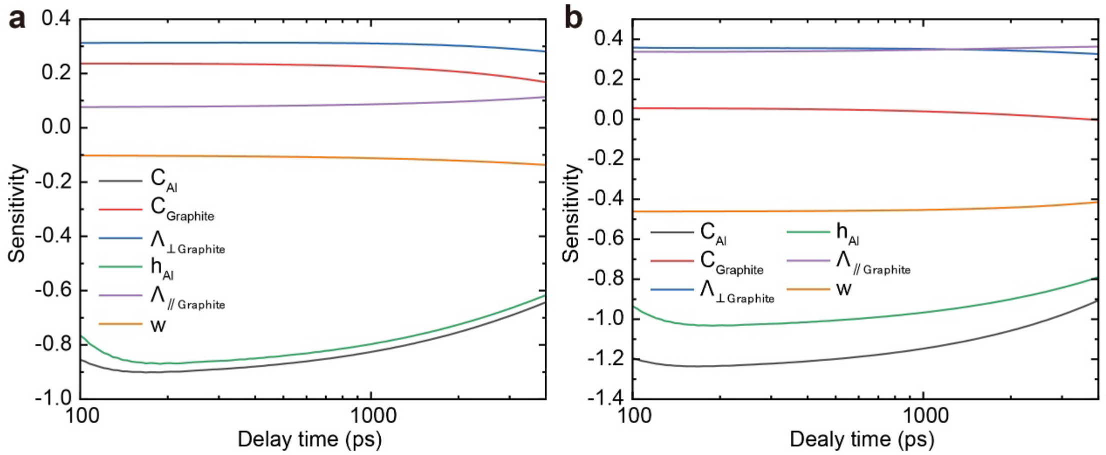

Supplementary Fig. 15 | Sensitivity and uncertainty analysis of TDTR method by taking PJHIC-graphite film as an example. (a) Sensitivity to parameters in TDTR measurements of the cross-plane thermal conductivity of graphite. (b) Sensitivity to parameters in TDTR measurements of the in-plane thermal conductivity of graphite.

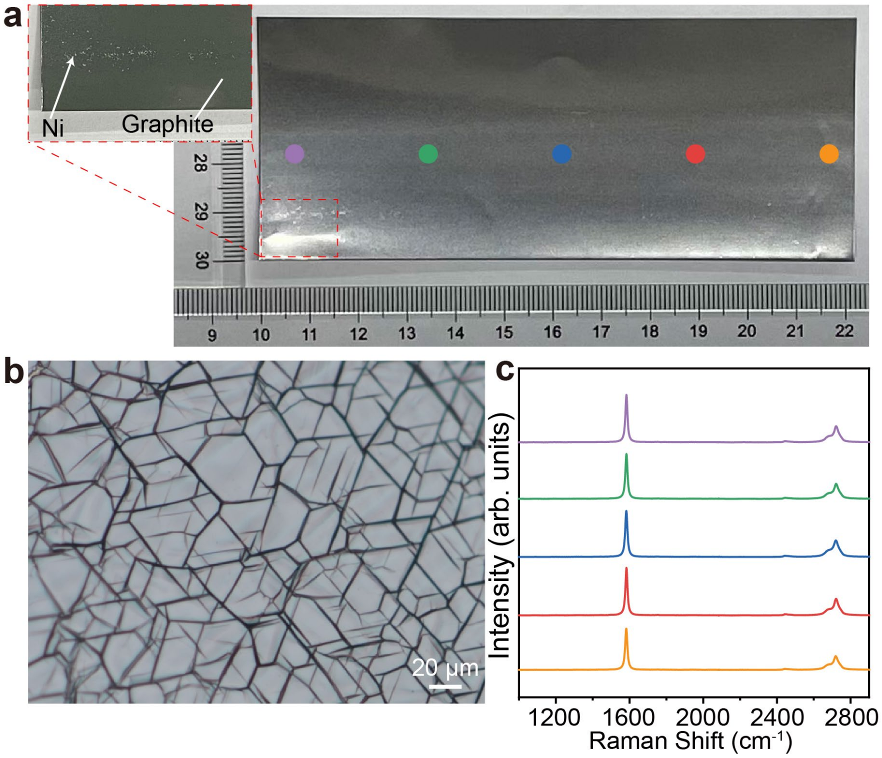

Supplementary Fig. 16 | Synthesis of 12 cm × 5 cm graphite film grown on a Ni foil. (a) Optical image. (b) Typical optical microscopy image of the film. (c) Raman spectra of the graphite film.

Supplementary Reference

1 Lander, J. J., Kern, H. E. & Beach, A. L. Solubility and Diffusion Coefficient of Carbon in Nickel: Reaction Rates of Nickel-Carbon Alloys with Barium Oxide. *J. Appl. Phys.* **23**, 1305-1309 (1952). https://doi.org/10.1063/1.1702064

2 Wang, L., Gao, J. & Ding, F. Application of crystal growth theory in graphene CVD nucleation and growth. *Acta Chim. Sin.* **72**, 345-358 (2014).

3 Gao, J. F., Yip, J., Zhao, J. J., Yakobson, B. I. & Ding, F. Graphene Nucleation on Transition Metal Surface: Structure Transformation and Role of the Metal Step Edge. *J. Am. Chem. Soc.* **133**, 5009-5015 (2011). https://doi.org/10.1021/ja110927p

4 Lahiri, J., Miller, T., Adamska, L., Oleynik, I. I. & Batzill, M. Graphene Growth on Ni(111) by Transformation of a Surface Carbide. *Nano Lett.* **11**, 518-522 (2011). https://doi.org/10.1021/nl103383b

5 Liu, C. *et al.* Kinetic modulation of graphene growth by fluorine through spatially confined decomposition of metal fluorides. *Nat. Chem.* **11**, 730-736 (2019). https://doi.org/10.1038/s41557-019-0290-1

6 Huang, M. *et al.* Highly Oriented Monolayer Graphene Grown on a Cu/Ni(111) Alloy Foil. *ACS Nano* **12**, 6117-6127 (2018). https://doi.org/10.1021/acsnano.8b02444

7 Cahill, D. G. Analysis of heat flow in layered structures for time-domain thermoreflectance. *Rev. Sci. Instrum.* **75**, 5119–5122 (2004). https://doi.org/10.1063/1.1819431
